# Conformation‐Selective Binding Domains of Intramolecular Chemically Induced Protein Dimerisation Systems

**DOI:** 10.1002/anie.9707369

**Published:** 2026-10-02

**Authors:** Zhenling Cui, Zhong Guo, Yi Jin Liew, Alessandro T. Caputo, Maria M. Fiorito, Cagla Ergun Ayva, Roxane Mutschler, Mayra Barrios Leguarca, Sophia Newton, Gia Nghi Lam, Robert Speight, Brett McWhinney, Yanulla Essie Georges, Carel Pretorius, Jacobus P.J. Ungerer, Kirill Alexandrov

**Affiliations:** ^1^ ARC Centre of Excellence in Synthetic Biology Brisbane Queensland Australia; ^2^ Centre For Agriculture and the Bioeconomy Queensland University of Technology Brisbane Queensland Australia; ^3^ School of Biology and Environmental Science Queensland University of Technology Brisbane Queensland Australia; ^4^ Advanced Engineering Biology Future Science Platform Commonwealth Scientific and Industrial Research Organisation (CSIRO) Dutton Park Queensland Australia; ^5^ CSIRO Health & Biosecurity Westmead New South Wales Australia; ^6^ CSIRO Manufacturing Research Unit, Research Way Clayton Victoria Australia; ^7^ Department of Chemical Pathology Pathology Queensland Brisbane Queensland Australia; ^8^ Faculty of Health and Behavioural Sciences The University of Queensland Brisbane Queensland Australia

**Keywords:** artificial protein‐binding domains, chemically induced dimerisation, conformation‐selective binding domains, mRNA display, protein biosensors

## Abstract

Chemically induced protein dimerisation (CID) is a widely used approach for detecting and actuating small‐molecule‐protein interactions. Here, we demonstrate that CID systems can be used as building blocks for higher‐order ligand‐controlled protein systems. To this end, we converted classical FK506‐ and cyclosporine A‐controlled CIDs into single‐chain pseudo‐allosteric proteins. These systems displayed 15‐ and 50‐fold gains in ligand affinity compared with the parental components operating in trans. Using these fusion proteins as baits in mRNA display, we identified FN3con binding domains that recognise the holo state of each bait with subnanomolar affinity and >100‐fold selectivity over the apo form. Integration of the binding domains and intramolecular CIDs into two‐component enzymatic protein biosensors enabled quantification of the cognate ligands with low‐nanomolar limits of detection (LODs). ELISA assays based on the developed systems displayed mid‐picomolar LODs and accurately quantified FK506 and cyclosporine A in unprocessed blood samples from transplant patients with accuracy comparable to that of LC‐MS/MS. This work establishes a generalisable strategy for harnessing the affinity gains of intramolecular CID systems to create gain‐of‐signal assays for small molecules and ions.

## Graphical abstract

Intramolecular chemically induced dimerisation transforms FK506‐ and cyclosporine A‐responsive protein pairs into high‐affinity pseudo‐allosteric switches. Holo‐selective binders isolated by mRNA display convert these switches into gain‐of‐signal biosensors and ultrasensitive ELISAs, enabling direct quantification of both immunosuppressants in unprocessed patient blood with accuracy comparable to LC–MS/MS and revealing a general route to small‐molecule detection.

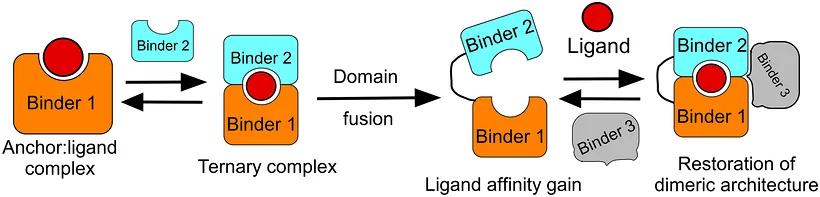

## 1 Introduction

The components of biological systems differ greatly in size and complexity. Despite the vast size difference between ions and biological polymers such as proteins and nucleic acids, these entities assemble seamlessly into complex functional networks. Constructing artificial functional systems composed of chemicals entities at different scales is a key objective of synthetic biology and bioengineering. Chemically induced dimerisation (CID) systems have been utilised extensively to translate small‐molecule interactions with proteins into changes in protein proximity, local concentration and, by extension, activity (Figure 1A). Natural protein dimerisation systems, such as rapamycin‐controlled FK506‐binding protein 12 (FKBP12):FKBP‐rapamycin‐binding domain (FRB) heterodimers or auxin‐controlled transport inhibitor response 1 (TIR1):auxin/indole‐3‐acetic acid protein (AID) heterodimers, have been used extensively as research and bioengineering tools [1]. Subsequently, molecular display systems have been used to construct artificial CID systems starting from either a pre‐existing protein‐small‐molecule complex or a small molecule alone [2, 3, 4]. More recently, machine‐learning approaches have been used to design artificial small‐molecule‐binding proteins that were subsequently used as templates for constructing fully artificial CID systems [5].

**FIGURE 1:**
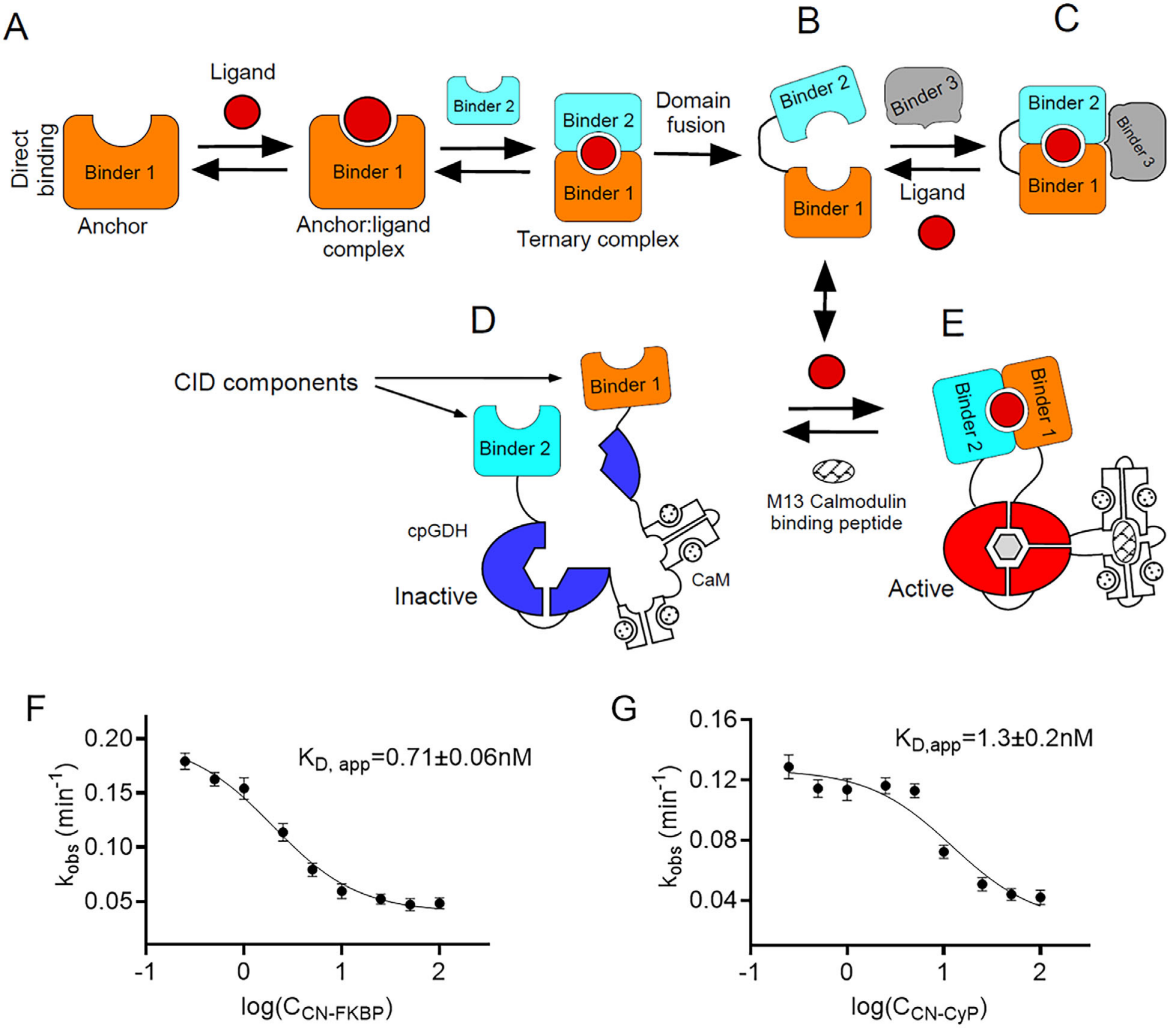
Conversion of an intermolecular chemically induced dimerisation (CID) system into an intramolecular CID and restoration of a two‐component architecture through selection of a conformation‐specific binder. (A) Schematic of ligand‐induced protein dimerisation that forms a CID system. (B) Conversion of an intermolecular CID system into an intramolecular system by fusing its subunits into a single polypeptide. (C) Recognition of the intramolecular CID by a conformation‐selective binder. (D) Schematic of single‐component circularly permuted glucose dehydrogenase‐calmodulin (cpGDH‐CaM) FK506 (tacrolimus) and cyclosporine A (CsA) biosensors controlled by the cognate ligands and M13 calmodulin‐binding peptide (CaM‐BP) [10]. cpGDH‐CaM FK506 biosensor is a fusion protein of CN‐cpGDH‐CaM‐FKBP, while cpGDH‐CaM CsA biosensor is a fusion protein of CN‐cpGDH‐CaM‐cyclophilin. (E) Concomitant binding of the ligand and M13 CaM‐BP results in rearrangement of the system and increased glucose dehydrogenase activity [10]. Panels B, D and E illustrate how the cpGDH‐CaM FK506 and CsA biosensors are used to assess the affinity of intramolecular CIDs. (F) Fit of data from experiments in which a 2.5 nM solution of the cpGDH‐CaM FK506 biosensor was titrated with increasing concentration of CN‐FKBP fusion protein in the presence of 10 nM FK506 and 250 nM M13 CaM‐BP. Following pre‐incubation of the components for 60 min, the reaction was initiated by addition of glucose, phenazine methosulfate (PMS), and 2, 6‐dichlorophenolindophenol (DCPIP), and the decrease in absorbance was monitored at 600 nm. Fitting the *k*_obs_ values yielded an apparent *K*_D_ value (*K*_D_, app) of 0.71 nM for the interaction of CN‐FKBP with FK506. (G) As in (F), but using the cpGDH‐CaM CsA biosensor, CN‐Cyp fusion protein, and CsA. Fitting the data yielded an apparent *K*_D_ value of 1.3 nM for the interaction of CN‐Cyp with CsA. The plots show mean *k*_obs_ values from three independent biological experiments.

It is well established that increasing the valency of ligand‐protein interactions can increase the overall affinity and selectivity of a system [6]. In their foundational work, Koide and colleagues demonstrated that linking the components of a CID system increases its affinity for cognate ligands by more than three orders of magnitude [4]. However, this gain comes at the cost of the CID system's ability to control molecular proximity, as it effectively becomes an intramolecular pseudo‐allosteric system. In this study, we set out to test whether it is possible to harness the affinity gains of intramolecular CID systems while restoring their two‐component architecture through the development of conformation‐selective binding domains (Figure 1B,C). We demonstrate the construction of coupled intra‐ and inter‐molecular CID systems and their use as biosensors for the macrocyclic compounds FK506 and cyclosporine A (CsA).

## 2 Results and Discussion

We expected that converting the intermolecular CIDs controlled by FK506 and CsA into single polypeptides would increase their affinity for these ligands through cooperative effects and reduced ligand dissociation rates [6]. To test this experimentally, we analysed the structures of canonical CID systems based on calcineurin A/B (CN) in complex with FKBP12 or cyclophilin (Cyp), stabilised by FK506 and CsA, respectively (PDB: 1TCO and 1MF8) [7, 8]. The topology of these complexes guided the design of linker sequences connecting CN to FKBP and CN to Cyp in continuous open reading frames (Figure S1A,B). Genes encoding the CN‐FKBP and CN‐Cyp open reading frames were cloned into an *Escherichia coli* expression vector in frame with an N‐terminal His tag, a 15‐amino‐acid AviTag and a protease cleavage site (Table S1). Biotinylation of the AviTag occurred during co‐expression of the fusion proteins with the biotin ligase BirA in *E. coli* [9]. The recombinant proteins were purified by Ni‐NTA affinity chromatography and size‐exclusion chromatography.

To test whether the resulting fusion proteins, CN‐FKBP and CN‐Cyp, retained the ability to bind their cognate ligands, we used previously developed enzymatic protein biosensors for FK506 and CsA [10]. These single‐component circularly permuted glucose dehydrogenase calmodulin chimeras (cpGDH‐CaM) FK506 and CsA biosensors are activated by ligand binding and produce a time‐dependent decrease in absorbance at 600 nm (Figure 1D,E). We formulated a competitive assay in which we monitored biosensor activity in the presence of varying concentrations of CN‐FKBP or CN‐Cyp fusion protein and the cognate ligand (Figure S2A,B). If a fusion protein binds the ligand, it competes with the biosensor for ligand binding, reducing the fraction of ligand‐bound active biosensor and thereby decreasing the observed reaction rate. Fitting the data yielded apparent *K*_D_ values of 0.7 ± 0.06 nM for FK506 binding to CN‐FKBP and 1.3 ± 0.23 nM for CsA binding to CN‐Cyp (Figure 1F,G). These values represent 15‐ and 50‐fold gains in affinity, respectively, compared with the CID components in trans (11 nM and <70 nM, respectively) [11, 12].

The substantial gain in ligand affinity in intramolecular CIDs came at the cost of losing the dimeric architecture, making the ligand‐binding event more difficult to actuate. We hypothesised that the heterodimeric architecture could be restored by developing a protein‐binding domain capable of differentiating the apo and holo forms of the CN‐FKBP and CN‐Cyp fusion proteins (Figure 1B,C). To do this, we used a previously reported mRNA‐display platform in which the linkage between the coding mRNA and its protein product is maintained through a covalently linked puromycin molecule [13].

We utilised a monobody library based on the previously described FN3con domain, a small, disulfide‐free β‐sandwich scaffold with optimised electrostatic and hydrophobic properties (Table S2) [14]. We immobilised biotinylated CN‐FKBP fusion protein on streptavidin‐coated Dynabeads and used the beads to pre‐clear an FN3con library. The resulting library was then selected against fusion‐protein‐bound Dynabeads saturated with the cognate ligand (Figure S3A). During bead washing, FK506 was added to the wash buffer to prevent complex dissociation. For counter‐selection, the apo form of CN‐FKBP was added to the wash buffer. Outputs from rounds 1, 4, 5 and 6 were translated in vitro using the PUREfrex system and used as inputs in polyclonal ELISA assays against plate‐immobilised CN‐FKBP or CN‐FKBP:FK506 complexes. These experiments revealed complex‐specific binder enrichment in round 5 that was maintained in round 6 (Figure S3B,C).

We subsequently performed five rounds of mRNA‐display selection against the immobilised CN‐Cyp:CsA complex. The apo form of the CN‐Cyp fusion protein was used for counter‐selection. ELISA assays demonstrated substantial enrichment of binders against the CN‐Cyp:CsA complex in rounds 4 and 5 (Figure S3D,E). We observed some cross‐reactivity with apo CN‐Cyp, but, given the much stronger signal for the positive control, we proceeded with the selection.

NGS analysis of binder genes selected against both targets revealed that the final clone pools contained approximately 17 131 and 107 653 unique binder sequences for the FK506‐ and CsA‐controlled systems, respectively (Text S1, Tables S3 and S4, and Figures S4–S7).

To select the most promising binders for biophysical characterisation without purifying individual clones, we used a combination of ELISA and biosensor‐based assays that could be performed on bacterial supernatants (Text S2 and Figures S8–S12). Guided by this initial characterisation, we expressed and purified to near homogeneity the CN‐FKBP:FK506 binder fB1 and the CN‐Cyp:CsA binders cB1, cB6 and cG03, each tagged with a calmodulin‐binding peptide (CaM‐BP) (Table S5 and Figure S13). Pull‐down assays confirmed that fB1‐CaM‐BP and cB6‐CaM‐BP selectively recognise the holo but not the apo forms of their respective fusion proteins (Figure 2A,B and S14A). In biolayer interferometry experiments, fB1‐CaM‐BP showed no detectable binding to the apo form of CN‐FKBP but bound strongly to the CN‐FKBP:FK506 complex, with a *K*_D_ of 0.35 ± 0.008 nM, an association rate (*k*_on_) of 4.7 ± 0.03 × 10^5^ M^−1^ s^−1^ and a dissociation rate (*k*_off_) of 1.40 ± 0.03 × 10^−4^ s^−1^ (Figure S14B). Similarly, cB6‐CaM‐BP selectively bound to the CN‐Cyp:CsA complex (*K*_D_ = 9.7 ± 0.5 nM; *k*_on_ = 9.6 ± 0.02 × 10^4^ M^−1^ s^−1^; *k*_off_ = 8.5 ± 0.2 × 10^−4^ s^−1^; Figure S14C) but showed no measurable binding to apo CN‐Cyp.

**FIGURE 2:**
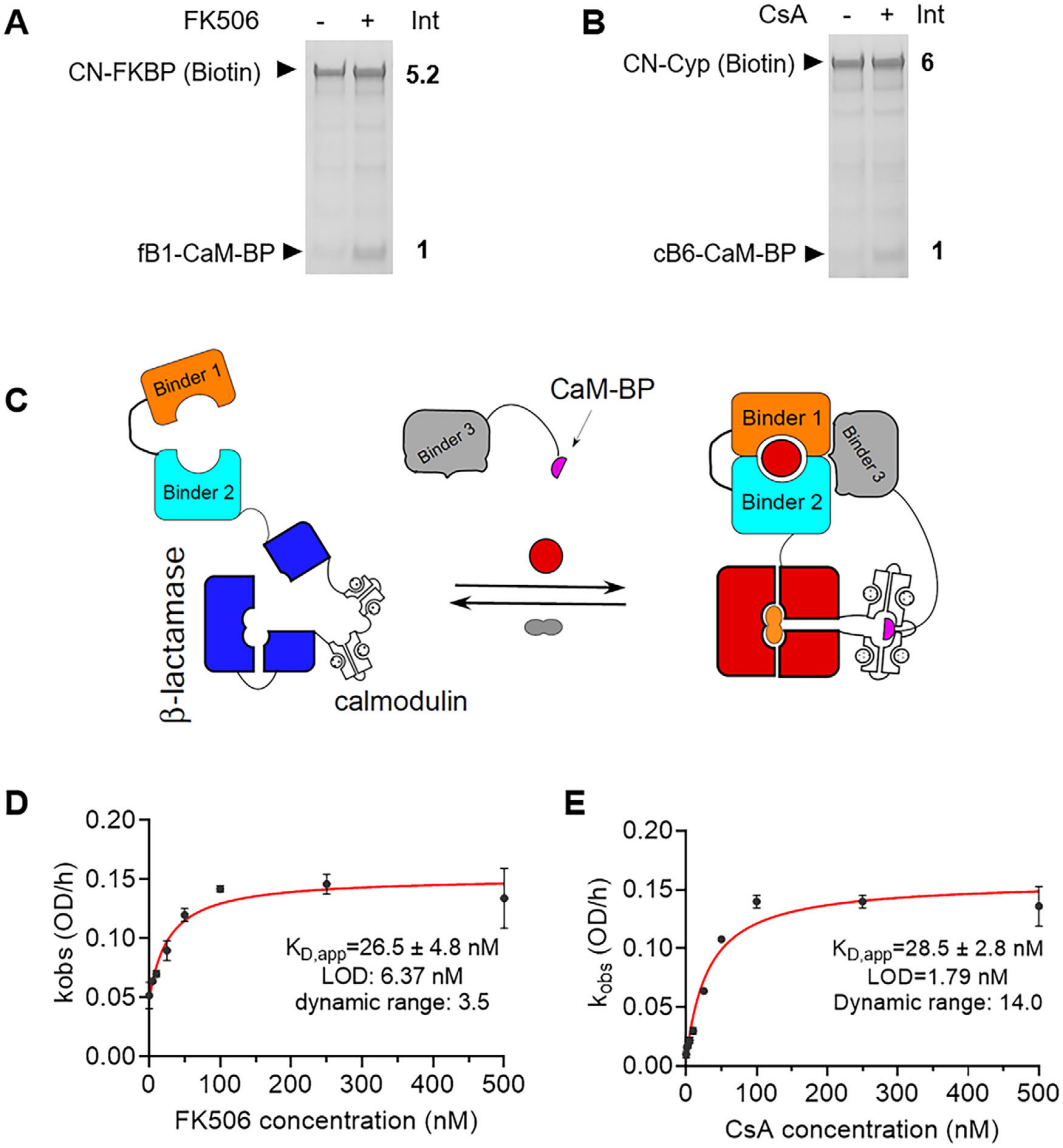
Specificity of conformation‐selective binders towards protein‐macrocyclic‐peptide complexes and their use in the construction of two‐component protein biosensors. (A) SDS‐PAGE analysis of a pull‐down assay in which biotinylated CN‐FKBP was incubated with purified fB1‐CaM‐BP in the absence or presence of FK506. Densitometric analysis showed that the band‐intensity ratio of fB1‐CaM‐BP (16 kDa) to CN‐FKBP (82 kDa) was approximately 1:5.2 in the presence of FK506. After correction for molecular weight, this corresponds to an estimated molar ratio of approximately 1:1, consistent with one fB1‐CaM‐BP molecule binding to one CN‐FKBP fusion protein. (B) SDS‐PAGE analysis of a pull‐down assay in which biotinylated CN‐Cyp was incubated with purified cB6‐CaM‐BP in the absence or presence of CsA. Densitometric analysis showed that the band‐intensity ratio of cB6‐CaM‐BP (16 kDa) to CN‐Cyp (89 kDa) was approximately 1:6 in the presence of CsA. After correction for molecular weight, this corresponds to an estimated molar ratio of approximately 1:0.93 for CN‐Cyp, consistent with near 1:1 binding between CN‐Cyp and cB6‐CaM‐BP. (C) Schematic representation of the two‐component biosensor architecture. The first component (biosensor core) comprises β‐lactamase (BLA) fused to CaM and the ligand‐binding receptor module, CN‐FKBP for FK506 or CN‐Cyp for CsA. The second component comprises a conformation‐selective binder fused to a CaM‐BP. Ligand binding generates the receptor conformation recognisably by the binder, recruiting BP to CaM and thereby allosterically activating BLA‐catalysed substrate hydrolysis. (D) Nonlinear regression fit of *k*_obs_ obtained by titrating FK506 into a solution containing 50 nM BLA‐CaM‐CN‐FKBP and 250 nM fB1‐CaM‐BP, yielding an apparent *K*_D_ of approximately 27 nM. The linear phase of each reaction was used to derive *k*_obs_ values, which were plotted against FK506 concentration and fitted to determine the apparent *K*_D_. (E) Same as in D but by titrating CsA into a solution containing 35 nM BLA‐CaM‐CN‐Cyp and 175 nM cB6‐CaM‐BP, yielding an apparent *K*_D_ of 29 nM. The *k*_obs_ and apparent *K*_D_ values shown in the plot are presented as mean ± SD from two independent experiments. The limit of detection (LOD) was calculated from the linear range of the titration curve as LOD = 3 × SD/S, where SD is the standard deviation of the response and S is the slope of the calibration curve.

To assess the performance of the conformation‐selective binders identified in our screen in two‐component biosensors based on allosterically regulated β‐lactamase (BLA), we titrated a solution of BLA‐CaM‐CN‐FKBP fusion protein and fB1‐CaM‐BP with increasing concentrations of FK506 in the presence of a chromogenic β‐lactamase substrate (Figure 2C and Figure S15A). In this system, FK506 binds to CN‐FKBP of the sensor core (BLA‐CaM‐CN‐FKBP) and triggers a conformation that is recognised by fB1, promoting association of the fused CaM‐binding peptide with CaM of the sensor core and thereby allosterically activating BLA‐catalysed hydrolysis of the chromogenic substrate. The dose‐dependent increase in the observed reaction rate (*k*_obs_), derived from the initial slopes of the absorbance traces, were fitted to a nonlinear one‐site binding model, yielding an apparent *K*_D_ of 27 ± 5 nM and a 3.5‐fold dynamic range (Figure 2D). We repeated this analysis using BLA‐CaM‐CN‐Cyp and cB6‐CaM‐BP with varying concentrations of CsA (Figure S15B), which yielded a *K*_D,app_ of 29 ± 3 nM and a dynamic range of 14‐fold (Figure 2E). We confirmed that, in the absence of the conformation‐selective binder‐BP component, the BLA‐CaM‐CN‐FKBP and BLA‐CaM‐CN‐Cyp sensor cores alone do not form functional ligand‐responsive sensors (Figure S15C,D). We further investigated the tolerance of the FK506 and CsA two‐component biosensors to different pH and temperature conditions. Although both sensors showed highest activity around pH 6.0, this condition also produced higher background; the best signal‐to‐noise ratio was observed around pH 7.0, close to the assay condition used throughout this study (Figure S15E,F). Temperature testing showed that the FK506 two‐component sensor retained activity at 4°C–37°C but lost most activity at 50°C or above, whereas the CsA two‐component sensor retained greater activity up to 37°C before activity was largely lost at higher temperatures (Figure S15G,H). Both sensors displayed high selectivity for their cognate ligands and produced no detectable response to the non‐cognate ligands or rapamycin (Figure S16).

As our attempts to obtain crystal structures of the engineered ternary complexes were unsuccessful, we modelled their structures using AlphaFold (Text S3) [15, 16, 17]. AlphaFold predictions of the holo CN‐FKBP:FK506 complex closely matched the crystallographic non‐fusion holo structure (PDB 1TCO). Modelling of fB1 binding to holo CN‐FKBP:FK506 produced a range of closely related conformations in which the binder associated with an FK506‐induced composite epitope formed by CN and FKBP (Figure S17). In contrast, predictions of the CN‐Cyp:CsA fusion structure had low confidence and deviated from the complex topology observed in the native ternary complex (PDB 1MF8).

Although the developed CsA biosensors were sufficiently sensitive to detect the drug in biological samples, the clinical concentration of FK506 is below the *K*_D,app_ value of the biosensor; therefore, an assay based on this biosensor is unlikely to have diagnostic utility. We, therefore, tested whether the developed binding domains could be deployed in alternative assay formats that would allow detection of both compounds in clinical samples. To this end, we modified the ELISA assay originally developed to analyse selected FN3con binders to make it ligand‐limited (Figure 3A). In the modified format, binding of the ligand to surface‐attached CN‐Cyp is detected using a panel of binders, among which 3Flag‐cB1‐CaM‐BP exhibited the best performance. This assay yielded an apparent *K*_D_ of 0.92 ± 0.1 nM and an LOD of 130 pM for CsA (Figure 3B; Figures S18 and S19A). This LOD is 640‐fold below the lower end of the therapeutic concentration range for CsA (83–333 nM) [18]. We compared the developed assay with a similarly configured ELISA based on the interaction of the individual CID components, in which surface‐immobilised Cyp was used for ligand capture and 3Flag‐tagged CN served as the reporter, and obtained an apparent *K*_D_ of 56.9 ± 5.4 nM (Figure S20). These results demonstrate that coupling the intermolecular CIDs produced an approximately 60‐fold gain in apparent affinity. This is consistent with the gain in ligand affinity of the CID fusion proteins compared with their counterparts in trans (Figure 1F,G).

**FIGURE 3:**
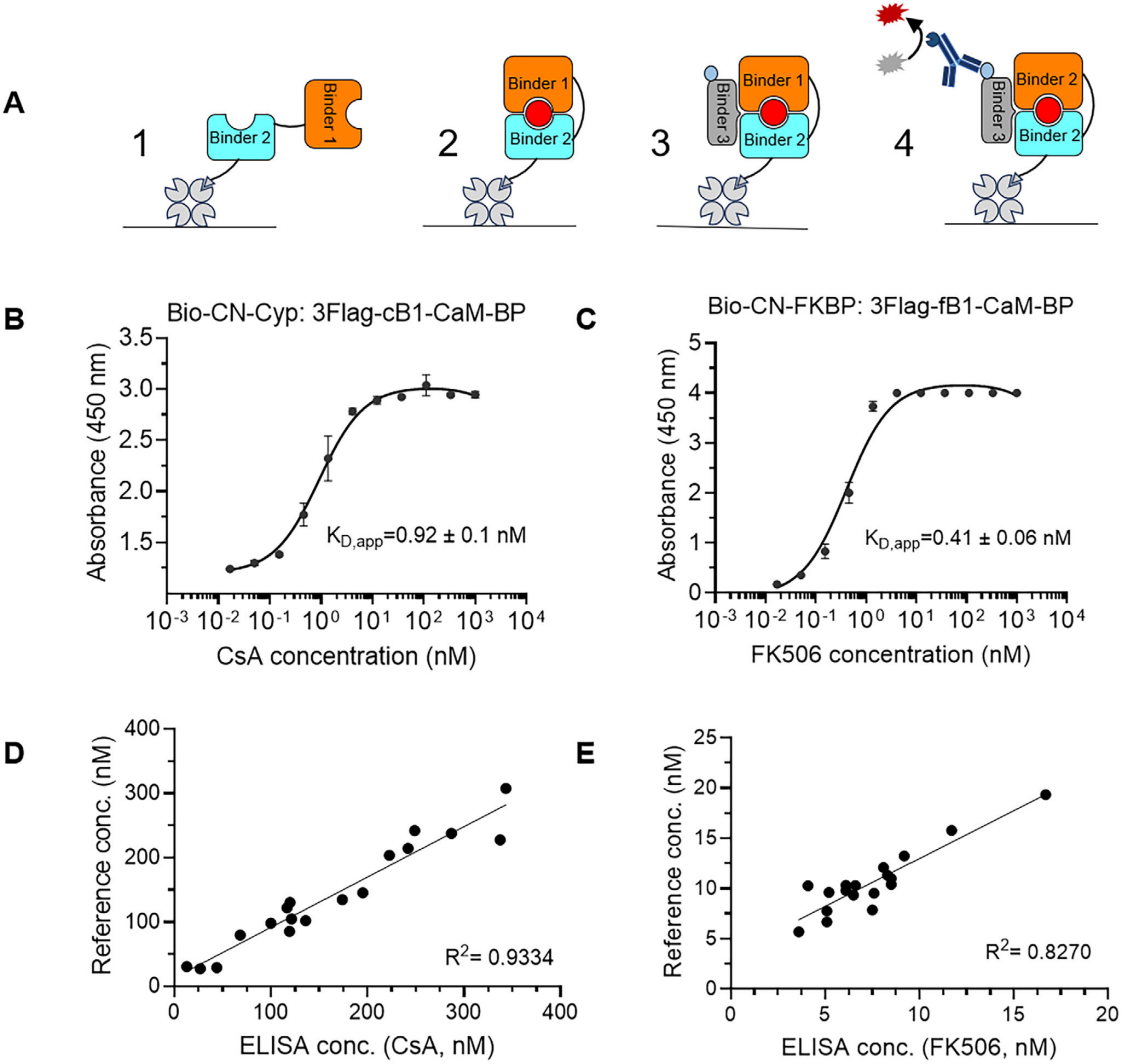
CsA and FK506 quantification using CID‐based ELISA assays. (A) Schematic of the indirect sandwich ELISA based on coupled intra‐ and inter‐molecular dimerisation systems. Streptavidin‐coated plates are incubated with a pre‐equilibrated mixture of biotinylated CN‐Cyp or CN‐FKBP and samples containing CsA or FK506. The resulting holo complexes recruit the Flag‐tagged FN3con binder. Detection is performed using an HRP‐conjugated anti‐Flag antibody and TMB substrate. (B) ELISA absorbance values plotted against CsA concentration using biotinylated CN‐Cyp and 3Flag‐cB1‐CaM‐BP, resulting in an apparent *K*_D_ of 0.92 nM. (C) As in (B), but using biotinylated CN‐FKBP, the fB1‐CaM‐BP binding domain and FK506 as the titrant, yielding an apparent *K*_D_ of 0.41 nM. Data are presented as mean ± SD from three replicates. (D) Comparison of CsA concentrations in blood samples from transplant patients determined by ELISA (x‐axis) with those determined by LC‐MS/MS (*y*‐axis). (E) Comparison of FK506 concentrations in blood samples from transplant patients determined by ELISA (*x*‐axis) with those determined by LC‐MS/MS (*y*‐axis).

Similarly, using surface‐immobilised CN‐FKBP as the bait and 3Flag‐tagged fB1‐CaM‐BP as the reporter, we obtained an apparent *K*_D_ for FK506 of 0.41 ± 0.06 nM, with a limit of detection (LOD) of 50 pM (Figures 3C and S19B). This LOD is approximately 120‐fold below the lower end of the therapeutic concentration range for FK506 (6‐24 nM) [19]. When the CID components were tested in trans in the ELISA assay, we obtained an apparent *K*_D_ of 4.5 ± 0.6 nM. Thus, coupling intra‐ and intermolecular CIDs produced an apparent affinity gain of approximately 10‐fold (Figure S20).

We next tested the compatibility of the developed ELISA assays with blood samples by performing the assays in the presence of 5 µL of whole blood. Using CN‐Cyp and its cognate binder, 3Flag‐cB1‐CaM‐BP, we determined the assay's apparent *K*_D_ for CsA to be 2.0 nM. The twofold decrease in affinity compared with the buffer‐only condition indicates mild matrix interference (Figure S21). The FK506 ELISA performed in the presence of whole blood displayed an apparent *K*_D_ of 1.04 nM, also indicating mild matrix interference (Figure S21). Using the CsA ELISA, we analysed blood samples from transplant patients undergoing CsA therapy. By adding 0.7 µL of blood to each ELISA, we reliably quantified CsA in these samples, achieving a correlation coefficient of 0.93 relative to values determined by LC‐MS/MS (Figure 3D). Subsequently, using 4 µL of whole blood per assay, we quantified FK506 with a correlation coefficient of 0.83 relative to LC‐MS/MS results (Figure 3E). To our knowledge, this represents a rare demonstration of accurate quantification of CsA and FK506 directly in whole blood without organic extraction of the drugs.

In this study, we explored the construction and application of coupled intra‐ and intermolecular protein dimerisation systems. We converted two CID systems controlled by the immunosuppressive drugs FK506 and cyclosporine A into single‐chain fusion proteins. This resulted in up to a 50‐fold increase in cognate‐ligand affinity, likely arising from reduced overall dissociation rates due to the presence of both ligand‐binding domains within the same polypeptide. Using mRNA display, we isolated FN3con binding domains that efficiently discriminate between the apo and holo forms of the fusion proteins. To facilitate the identification of such binders, we combined biosensor‐based assays with NGS and Sanger sequencing. Our results show that, whereas FK506 biosensor screening confirmed the top binder predicted by NGS, CsA biosensor screening uncovered additional functional binders that were not detected by either NGS or Sanger sequencing. This finding underscores the value of biosensor‐based high‐throughput screening as a complement to sequencing‐based analyses, particularly for identifying lower‐frequency binders.

The lead binder clones fB1 and cB6 exhibited subnanomolar to low‐nanomolar affinities and >100‐fold selectivity for the holo form of the intramolecular CID. Importantly, AI‐based structural modelling rationalised the mechanism of fB1 selectivity, revealing a ligand‐induced composite epitope spanning Calcineurin A and FKBP that cannot form in the apo ensemble. This echoes our previous findings on binder interactions with newly formed CID interfaces and highlights the broader principle that small molecules can stabilise unique interdomain structures that create novel epitopes accessible to engineered binders [20].

The coupled dimerisation systems were used to construct two‐component biosensors in which ligand‐induced scaffolding of the components activated allosteric reporter domains. These biosensors had double‐digit nanomolar affinities for the drugs in homogeneous‐solution assays. Although the assay sensitivity was sufficient to quantify cyclosporine A in clinical samples, there was little prospect that the same could be achieved for FK506, which is administered at a much lower concentration. This could potentially be overcome by affinity maturation of the developed binders to increase their affinity and, by extension, improve the biosensor's LOD. As an alternative, we tested the utility of the coupled dimerisation systems in ELISA assays. These assays achieved low‐nanomolar *K*_D_ values and subnanomolar LOD for CsA and FK506, representing a substantial improvement over what could be achieved with the parental CIDs. We accurately quantified both cyclosporine A and FK506 in clinical samples, demonstrating the practical utility of the developed assays. Thus, the combination of intramolecular CID systems and conformation‐selective binding domains can be used to create accurate gain‐of‐signal ELISA assays for small molecules. The sensitivity gains of these assays were consistent with the affinity gains of the chimeric fusions towards their ligands (50‐fold and 10‐fold, respectively).

Although this study used naturally occurring proteins, advances in AI‐driven protein design now enable the construction of fully artificial small‐molecule‐binding proteins and CID systems [21, 22, 23, 24, 25]. These advances substantially expand the repertoire of components available for constructing coupled intramolecular and intermolecular CID systems. In this study, we detected ligand‐induced structural changes in intramolecular CID systems using proximity‐based assays, including two‐component biosensors and ELISA. These structural changes could also be coupled directly to other sensing modalities, such as protein‐ or aptamer‐based biosensors. This enables the conversion of small‐molecule quantification assays from competitive, loss‐of‐signal formats to potentially more sensitive and robust gain‐of‐signal formats. Beyond analytical and diagnostic applications, these systems could serve as components of artificial signalling hubs that convert a single input into multiple outputs.

## Author Contributions

**Zhenling Cui**: conceptualization, investigation, writing – original draft, methodology, validation, visualization, writing – review and editing, formal analysis, data curation and supervision. **Zhong Guo**: conceptualization, methodology, formal analysis, data curation, supervision and resources. **Yi Jin Liew**: investigation, methodology and software. **Alessandro T. Caputo**: investigation and supervision. **Maria M Fiorito**: supervision, project administration, methodology. **Cagla Ergun Ayva**: conceptualization, investigation, methodology, visualization, formal analysis and data curation. **Roxane Mutschler**: investigation and validation. **Mayra Barrios Leguarca**: investigation and validation. **Sophia Newton**: methodology and investigation. **Gia Nghi Lam**: investigation. **Robert Speight**: conceptualization, funding acquisition and supervision. **Brett McWhinney**: methodology, validation, resources, data curation and investigation. **Yanulla Essie Georges**: investigation and resources. **Carel Pretorius**: conceptualization, methodology, validation, formal analysis and data curation. **Jacobus PJ Ungerer**: conceptualization, validation, methodology, data curation, resources. **Kirill Alexandrov**: conceptualization, investigation, funding acquisition, writing – original draft, methodology, validation, visualization, writing – review and editing, formal analysis, project administration, data curation, supervision and resources.

## Ethics Statement

Human blood samples were collected under approval from the Coronial and Public Health Sciences Human Ethics Committee (CPHS‐HEC), Pathology Queensland, Queensland Health (HREC/2026/CPHS/126933; HEC 26‐14; EC00305), with a waiver of informed consent. Administrative review approval was provided by the Queensland University of Technology Office of Research Ethics and Integrity (HE‐AdRev 2026‐11316‐30187).

## Conflicts of Interest

The authors declare no conflicts of interest.

## Supporting information



The supporting tables contain the protein and DNA sequences used in this study; NGS data from the naïve library and the FK506 and CsA selection campaigns; and summaries of potential binders identified by Sanger sequencing or biosensor assays. They also provide confidence scores and structural analyses from AlphaFold predictions. The supporting figures include schematic representations of the CN‐FKBP fusion protein and linker placements, hierarchical clustering and correlation heatmaps of binder‐sequence pools across selection rounds, sequence‐evolution trajectories, the workflow for candidate‐binder validation, assessment of biosensor activity using bacterial culture supernatants, functional analysis of the biosensor core, screening of top binders by biosensor assay, SDS‐PAGE analysis of purified binders, sensor‐specificity analyses and ELISA‐based quantification of FK506 and CsA concentrations. The authors have cited additional references within the Supporting Information [26‐32].**Supporting File 1**: anie75059‐sup‐0001‐SuppMat.docx.

**Supporting File 2**: anie75059‐sup‐0002‐SuppMat.xlsb.

## Data Availability

The data that supports the findings of this study are available in the Supporting Information of this article.
