# Supplementary material for: Conformation‐Selective Binding Domains of Intramolecular Chemically Induced Protein Dimerisation Systems
Source: Angew Chem Int Ed Engl. 2026 Oct 2;65(46):e9707369. doi: 10.1002/anie.9707369 (PMC13749266; doi:10.1002/anie.9707369)
Supplement: Supplementary file 1 [file ANIE-65-e9707369-s002.docx]

**Supplementary Information**

**Conformation-Selective Binding Domains of Intramolecular Chemically Induced Protein Dimerization Systems**

Zhenling Cui ^*[1][2][3][4]^, Zhong Guo ^[1][2][3]^, Yi Jin Liew ^[5]^, Alessandro T. Caputo ^[6]^, Maria M Fiorito ^[2][3]^, Cagla Ergun Ayva ^[2][3]^, Roxane Mutschler ^[2][3],^ Mayra Barrios Leguarca ^[2][3]^, Sophia Newton ^[6]^, Gia Nghi Lam ^[6]^, Robert Speight ^[4]^, Brett McWhinney ^[7][8]^, Yanulla Essie Georges ^[7][8]^, Carel Pretorius ^[7][8]^, Jacobus PJ Ungerer ^[7][8]^ and Kirill Alexandrov ^*[1][2][3]^

*Correspondence to: [zhenling.cui@qut.edu.au](mailto:zhenling.cui@qut.edu.au) and [kirill.alexandrov@qut.edu.au](mailto:kirill.alexandrov@qut.edu.au)

[1] ARC Centre of Excellence in Synthetic Biology, Brisbane, Queensland 4001, Australia

[2] Centre for Agriculture and the Bioeconomy, Queensland University of Technology, Brisbane, Queensland 4001, Australia

[3] School of Biology and Environmental Science, Queensland University of Technology, Brisbane, Queensland 4001, Australia

[4] Advanced Engineering Biology Future Science Platform, Commonwealth Scientific and Industrial Research Organisation (CSIRO), Dutton Park, QLD, Australia

[5] CSIRO Health & Biosecurity, Westmead, New South Wales 2145, Australia

[6] CSIRO Manufacturing Research Unit, Research Way, Clayton, VIC, 3168, Australia

[7] Department of Chemical Pathology, Pathology Queensland, Brisbane, Queensland 4006, Australia

[8] Faculty of Health and Behavioural Sciences, The University of Queensland, Brisbane, Queensland 4072, Australia

**Supplementary Figures**

**Supplementary Figure 1**. Models of the fusion proteins used in this study. **(A)** Ribbon representation of the CN-FKBP chimera in *the apo* form (left) and in complex with FK506, shown in orange in a ball-and-stick representation. Linker sequences connecting the subunits are shown as grey ribbons. CN is calcineurin A (CNA) and calcineurin B (CNB) fusion protein with a short GS linker. **(B)** As in (A), but showing the CN-Cyp fusion and its complex with cyclosporine A, shown in orange in a ball-and-stick representation. The structures of the apo fusion proteins were predicted using AlphaFold2, whereas those of the fusion-protein-drug complexes were predicted using the Protenix implementation of AlphaFold3. The predicted template modelling (pTM) and interface predicted template modelling (ipTM) scores for the CN-FKBP:FK506 complex were 0.85 and 0.98, respectively. The CN-Cyp:CsA complex had pTM = 0.64 and ipTM = 0.50.

**Supplementary Figure 2**. Representative real-time kinetic traces from competitive cpGDH-CaM FK506 and CsA biosensor assays used to assess ligand binding by the CN-FKBP and CN-Cyp fusion proteins. (A) Activity of the cpGDH-CaM FK506 biosensor in the presence of increasing concentrations of CN-FKBP fusion protein. The assay was performed as described in Figure 1F. 2.5 nM cpGDH-CaM FK506 biosensor was preincubated with varying concentrations of CN-FKBP, 10 nM FK506, 250nM M13 for 60 min. The reaction was then initiated by addition of glucose, electron mediated phenazine methosulfate (PMS) and 2,6-dichlorophenolindophenol (DCPIP), and the absorbance at 600 nm was monitored. (B) Activity of the cpGDH-CaM CsA biosensor in the presence of increasing concentrations of CN-Cyp. The assay was performed as described in Figure 1G.

Supplementary Figure 3. mRNA display selection of FN3con binders targeting CN-FKBP:FK506 and CN-Cyp:CsA complexes. (A) Schematic of the mRNA display workflow used to select conformation-specific binding domains that selectively recognize the holo forms of CN-FKBP and CN-Cyp from an FN3con domain library. The DNA library is transcribed into RNA (step 1) and then conjugated to a puromycin oligonucleotide linker (step 2). The mRNA-puromycin conjugate is used as a template for *in vitro* translation in the absence of release factors (step 3). In this context, the ribosome stalls at the stop codon and promotes formation of a covalent bond between puromycin and the nascent polypeptide chain. Following reverse transcription (step 4), the resulting cDNA/mRNA-puromycin-protein (mRPPD) conjugates are subjected to counter-selection against the apo-fusion-saturated matrix (step 5) and then to positive selection against the complex-saturated matrix (step 6). The selected genes are amplified by PCR (step 7) and used in the next selection round. (B,C) Analysis of mRNA display-selected binders against the CN-FKBP:FK506 complex by polyclonal ELISA. Cell-free protein synthesis (CFPS) reactions primed with cDNA outputs from rounds 1, 4, 5 and 6 were serially diluted and used as ELISA inputs. Binding to *apo* CN-FKBP (B) and holo CN-FKBP:FK506 (C) was detected using an HRP-conjugated anti-Flag antibody. (D,E) Analysis of binders selected against the CN-Cyp:CsA complex. ELISA signals were obtained from serially diluted CFPS reactions primed with cDNA outputs from rounds 1, 3, 4 and 5. Binding of *in vitro-produced* FN3con domains to CN-Cyp (D) and the CN-Cyp:CsA complex (E) was detected as in (B).

### Supplementary Text 1. Sequence analysis of putative holo-protein binding domains

NGS analysis of cDNA pools from the naïve library and successive selection rounds (round 6 for CN-FKBP:FK506 and round 5 for the CN-Cyp:CsA complex) revealed a progressive reduction in sequence diversity characteristic of selective enrichment (Supplementary Tables 3 and 4). In the naïve library, the proportion of unique sequences (R_unique/total_) exceeded 90%, whereas the frequency of the most abundant sequence (R_MAXfrequency/total_) was only approximately 0.4%, consistent with previous findings ^[26]^. For both campaigns, R_unique/total_ remained high (≥90%) through the first three selection rounds. A sharp drop in diversity was observed thereafter: for the CN-FKBP:FK506 selection campaign, diversity declined to 18% in round 4 and fell below 10% in rounds 5-6; for the CN-Cyp:CsA campaign, it decreased to 86% in round 4 and 33% in round 5. In the final rounds, 17,131 and 107,653 unique sequences remained for CN-FKBP:FK506 and CN-Cyp:CsA, respectively. Concurrently, R_MAXfrequency/total_ values were low in the early rounds but rose steeply to 61% for CN-FKBP:FK506 and 22% for CN-Cyp:CsA in the final rounds, reflecting the dominance of a few highly enriched binders.

Correlation analysis of sequence populations across selection rounds revealed three major clusters (Supplementary Figure 4). The first cluster contained the naïve-library replicates (naive-0a, -0b and -0c), together with CsA-1, FK506-1 and FK506-2, indicating that the early CN-Cyp:CsA and CN-FKBP:FK506 rounds were similar to the starting pool. The second cluster encompassed the mid-to-late CN-FKBP:FK506 rounds (FK506-3 to FK506-6), showing strong within-campaign convergence towards a distinct CN-FKBP:FK506-specific sequence population. Notably, CsA-2 did not fall within the first cluster but instead fell within the second, displaying high similarity to the CN-FKBP:FK506 selections and moderate similarity to CsA-3 through CsA-5, possibly reflecting shared selection pressure or cross-reactivity early in the CN-Cyp:CsA campaign. The third cluster comprised the late CN-Cyp:CsA rounds (CsA-3 to CsA-5), which occupied a distinct sequence space from both the CN-FKBP:FK506 selections and the naïve library. Overall, cross-campaign correlations were low, demonstrating that, despite targeting complexes sharing the same CN domain, each campaign enriched largely non-overlapping binders specific to either the CN-FKBP:FK506 or CN-Cyp:CsA complex, with the major divergence emerging after round 3.

To monitor sequence evolution and enrichment throughout the selection campaigns, we generated bump charts using the top 20 unique sequences from the first (R1) and final selection rounds (R6 for CN-FKBP:FK506 and R5 for CN-Cyp:CsA) as reference points. Bump-chart analysis highlighted differences in enrichment dynamics between the CN-FKBP:FK506 and CN-Cyp:CsA campaigns (Supplementary Figures 5 and 6). For CN-FKBP:FK506, several low-abundance sequences in round 1 (e.g., YRLGWGAD|GPGIRY|WDAIYWQT and VAQSYIPF|RAGHTR|SGVVYYA) rapidly increased in frequency between rounds 2 and 4, culminating in a dominant clone by round 6 (frequency per million (FPM) = 612,647, approximately 61%). Early-round leaders such as EPPPGPT|GSETS|NGAGEGPP declined sharply and disappeared from the top ranks, reflecting strong selection pressure and displacement of non-specific or low-affinity binders. By the final round, the pool was almost entirely dominated by a single sequence cluster, consistent with a classic selective sweep towards high-affinity CN-FKBP:FK506-specific binders (Supplementary Figure 5).

In contrast, the CN-Cyp:CsA campaign displayed slower convergence and sustained sequence diversity through the final round (Supplementary Figure 6). Dominant clones reached 227,073 FPM (22.7% of the pool), with multiple lineages from different clusters expanding in parallel. Several early-round sequences persisted into later rounds, indicating moderate affinity or specificity sufficient to withstand selection. The final pool retained representatives from multiple clusters (red, blue, green and orange), in contrast to the CN-FKBP:FK506 campaign, whose endpoint was dominated by a single cluster (red). These features suggest that the CN-Cyp:CsA selections underwent a more balanced and diversified enrichment process, potentially reflecting broader epitope tolerance and yielding a wider range of binders for downstream characterisation.

To validate the enrichment of top-performing sequences identified by NGS and obtain their physical DNA copies, cDNA pools from the final selection rounds were cloned into an *E. coli* expression vector in frame with an N-terminal His tag and a C-terminal calmodulin-binding peptide (CaM-BP); the resulting constructs were subsequently transformed into cloning and expression strains (Supplementary Figure 7). Sanger sequencing revealed multiple sequences recovered more than once that corresponded to the most highly enriched sequences in the NGS dataset, confirming their selection during the campaign (Supplementary Table 5). Comparison of the NGS and Sanger sequencing results indicated a practical detection threshold of approximately 50,000 FPM (approximately 5% of the pool), above which sequences had a high probability of being detected by Sanger sequencing, whereas sequences below 5% were rarely detected by this method.

**Supplementary Figure 4**. Hierarchical clustering and correlation heatmap of binder-sequence pools across selection rounds. Pairwise Pearson correlation coefficients were calculated from NGS-derived sequence frequencies of FN3con libraries selected against CN-FKBP:FK506 or CN-Cyp:CsA complexes, as well as the naïve input library. The heatmap displays correlation values from 0 (white) to 1 (dark purple), with clustering shown along both axes. Naïve replicates (naïve-0a-c) cluster tightly with the early CN-Cyp:CsA pool (CsA-1, corresponding to round 1 of the CN-Cyp:CsA binder selection campaign), whereas later rounds diverge into two distinct groups corresponding to CN-FKBP:FK506 selections (FK506-1 to FK506-6) and CN-Cyp:CsA selections (CsA-3 to CsA-5).

**Supplementary Figure 5. Sequence-evolution trajectories during CN-FKBP:FK506 binder selection.** Bump chart showing rank trajectories of enriched FN3con sequences across six rounds of mRNA display selection against the CN-FKBP:FK506 complex. Each line represents a unique peptide, coloured by cluster identity derived from sequence-similarity analysis (cluster IDs 0-4). The *y-axis* indicates sequence rank in each round, whereas the *x-axis* shows the panning round. Relative frequencies (fragments per million, FPM) are indicated to the right of each sequence in the first and final rounds.

**Supplementary Figure 6. Sequence-evolution trajectories during CN-Cyp:CsA binder selection.** The bump chart was constructed as described for Supplementary Figure 5 and shows rank trajectories of FN3con sequences across five rounds of mRNA display selection against the CN-Cyp:CsA complex.

**Supplementary** **Figure 7**. Schematic overview of the workflow for selection and validation of binders generated by mRNA display. cDNA from the final selection pools (round 6 for CN-FKBP:FK506 and round 5 for CN-Cyp:CsA) was cloned in frame with a calmodulin-binding peptide and transformed into *E. coli* TOP10. Two parallel routes were followed: in the first route (left), single colonies were selected for Sanger sequencing and plasmid minipreparation, transformed into *E. coli* BL21(DE3), and used for protein production, purification and testing. In the alternative route (right), miniprep-ed plasmids were transformed into *E. coli* BL21(DE3), single colonies were tested using the biosensor assay, and the hits were sequenced, followed by protein purification and functional testing. Approximately 90% of colonies from the cloning strain yielded valid sequences, whereas sequencing success decreased to 40-70% for the expression strain, likely because of plasmid quality or PCR artefacts (Supplementary Table 5).

### Supplementary Text 2. Biosensor-based evaluation of the enriched binder library

mRNA display selection campaigns result in >10^4^ unique binders, making it difficult to characterise the entire binder population (Supplementary Table 3). To streamline characterisation, we used previously reported biosensors based on artificial allosteric chimeras between calmodulin and the reporter enzyme TEM-1 β-lactamase (BLA) ^[27]^. This catalytic core is inactive in its *apo* state but regains catalytic activity upon binding to a calmodulin-binding peptide (CaM-BP). When ligand-binding domains are fused to this core and to CaM-BP, ligand-induced scaffolding stimulates reporter-domain activity (Figure 2C). Because of the high catalytic activity of β-lactamase, biosensor activity can be measured directly in *E. coli* supernatants using protein released from lysed biosensor-expressing *E. coli* cells (Supplementary Figures 8 and 9).

We constructed the CsA biosensor by cloning CN-Cyp fusion into a bacterial expression vector in frame with an N-terminal TEM-1 β-lactamase-calmodulin chimera switch module (BLA-CaM) and a C-terminal His tag ^[27]^. We expressed and purified BLA-CaM-CN-Cyp in *E. coli* and confirmed its functionality using wild-type M13 CaM-BP (Supplementary Figure 10). We then mixed it with supernatants from 48 clones expressing FN3con-CaM-BP fusions in the presence or absence of CsA. Supernatants from cE11, cG03, cH03 and cH04 showed clear ligand-mediated increases in β-lactamase activity (Supplementary Figure 11). Interestingly, clone cH04 (identified by Sanger sequencing) and clone cE11 matched top NGS binders (cB1-CaM-BP and cB6-CaM-BP), whereas cH03 and cG03 were not detected using either sequencing approach (Supplementary Table 5). This finding demonstrates that functional screening complements sequence-based analysis of the binder population and enables identification of low-frequency functional binders.

The FK506 biosensor core was constructed as a BLA-CaM-CN-FKBP chimera, and its functionality was confirmed by activation with wild-type M13 CaM-BP (Supplementary Figure 10). Supernatants from 20 expression clones carrying FN3con-CaM-BP constructs were incubated with recombinant BLA-CaM-CN-FKBP in the presence or absence of FK506. In these experiments, several clones showed a clear increase in activity upon FK506 addition (Supplementary Figure 12). Two such clones (fA09 and fD09) were sequenced, found to share the same sequence, and were also identified in the NGS data (fB1-CaM-BP; Supplementary Table 5).

**Supplementary Figure 8.** **Analysis of β-lactamase biosensor activity in supernatants of biosensor-producing *E. coli* cells.** (A) Analysis of β-lactamase activity in supernatants of *E. coli* BL21(DE3) cells expressing pET28a-2CaM-BLA in the presence (+) or absence (-) of M13 CaM-BP. Cells were grown in LB containing kanamycin, induced at OD600 approximately 0.8 with 0.3 mM IPTG, and incubated overnight at 18 °C. Untreated cell supernatants (5, 10, 20, 40 µL) or 20 µL of supernatant from cells subjected to three freeze-thaw cycles (20R) were assayed in a final volume of 200 µL containing 20 mM Tris (pH 7.2), 100 mM NaCl, 1 mM CaCl_2_ in the absence or presence of 200 nM M13. After 15 min incubation, the β-lactamase substrate UW154 was added to a final concentration of 50 µM, and absorbance at 520 nm was recorded. (B) Relative *k*_obs_ values of purified biosensor proteins, including wild-type BLA, 2CaM-BLA, 2CaM-BLA-2MTXVHH and 2CaM-BLA-2AmylaseVHH proteins described previously ^[28]^. The biosensor constructs, which contain two calmodulin domains inserted at distinct sites within BLA, retained robust activity. (C) SDS-PAGE analysis of culture supernatants from *E. coli* BL21(DE3) expressing the indicated biosensors: lanes 1 and 5, 2CaM-BLA; lanes 2 and 6, 2CaM-BLA-2MTXVHH; lanes 3 and 7, thermostable 2CaM-BLA-2MTXVHH mutant; and lanes 4 and 8, 2CaM-BLA-2AmylaseVHH. Lanes 1-4 correspond to supernatants from untreated cells, and lanes 5-8 correspond to supernatants from cells subjected to three freeze-thaw cycles. Asterisks indicate bands corresponding to the biosensor proteins.

**Supplementary Figure 9.** Relative activity of supernatants from *E. coli* cultures expressing the biosensor constructs (2CaM-BLA, 2CaM-BLA-2MTXVHH and 2CaM-BLA-2AmylaseVHH ) ^[28]^. (**A)** M13-induced activity of biosensor-containing supernatants from cells subjected to three freeze-thaw cycles. Clarified supernatants (20, 40 or 80 µL) were added to a 200 µL biosensor assay. Biosensor activity was normalised to that of the 80 µL supernatant from 2CaM-BLA-2AmylaseVHH, which exhibited the lowest activity. (**B**) Dynamic range, calculated as the ratio of biosensor activity in the presence versus the absence of M13 CaM-BP.

**Supplementary Figure 10**. Functional assessment of the BLA-CaM-CN-FKBP and BLA-CaM-CN-Cyp fusion proteins. Assays were performed using 25 or 50 nM of each fusion protein in the presence or absence of 200 nM M13 CaM-BP. Reactions were initiated by addition of the chromogenic β-lactamase substrate UW154, and absorbance was monitored at 520nm.

**Supplementary Figure 11**. Analysis of CN-Cyp:CsA-complex binders using biosensor assay. Supernatants (30 µL) from individual *E. coli* BL21 colonies expressing FN3con-CaM-BP constructs were added to a 200 µL biosensor assay containing 35 nM BLA-CaM-CN-Cyp in the absence (black) or presence (grey) of 1 µM CsA.

**Supplementary** **Figure 12**. Analysis of CN-FKBP:FK506-complex binders using the biosensor assay. Supernatants (30 µL) from individual *E. coli* BL21 colonies expressing FN3con-CaM-BP constructs were added to a 200 µL biosensor assay containing 35 nM BLA-CaM-CN-FKBP in the absence (black) or presence (grey) of 1 µM FK506.

**Supplementary Figure 13.** SDS-PAGE analysis of purified FN3con-CaM-BP proteins. The proteins were separated on a 4-12% gradient gel and imaged after staining with Coomassie Blue.

**Supplementary Figure 14.** Analysis of conformation-selective binders of intramolecular CIDs by pull-down assay and BLI. (A) Uncropped SDS-PAGE gel corresponding to Figure 2A,B. Lanes 1 and 2 show pull-downs of fB1-CaM-BP by immobilised biotinylated CN-FKBP in the absence and presence of FK506, respectively. Lanes 3 and 4 show pull-downs of cB6-CaM-BP by immobilised biotinylated CN-Cyp in the absence and presence of CsA, respectively. (B) BLI sensorgrams for binding of fB1-CaM-BP to the CN-FKBP:FK506 complex. Biotinylated CN-FKBP was pre-saturated with 2 µM FK506, immobilised on streptavidin biosensors, and titrated with fB1-CaM-BP at 0, 25, 50, 200, and 400 nM. Data were processed and analysed using ForteBio Data Analysis 9.0 software. After background subtraction, fitting of the binding curves yielded a K_D_ of 0.35 nM. (C) BLI sensorgrams for binding of cB6-CaM-BP to the CN-Cyp:CsA complex. The assay was performed as in (B), using biotinylated CN-Cyp pre-saturated with 2 µM CsA and titrated with cB6-CaM-BP. Fitting of the binding curves yielded a K_D_ of 9.7 nM.

**Supplementary Figure 15.** Activity analysis of two-component FK506 and CsA biosensors based on conformation-selective binders of intramolecular CIDs**. (A)** Real-time absorbance changes following addition of the chromogenic β-lactamase substrate UW154 to a solution containing 50 nM BLA-CaM-CN-FKBP, 250 nM fB1-CaM-BP and the indicated concentrations of FK506. (**B)** As in (A), but with CsA titrated into a solution containing 35 nM BLA-CaM-CN-Cyp and 175 nM cB6-CaM-BP. **(C)** Real-time absorbance changes of the FK506 biosensor in the presence or absence of fB1-CaM-BP and 200 nM FK506. **(D)** Real-time absorbance changes of the CsA biosensor in the presence or absence of cB6-CaM-BP and 200 nM CsA. **(E,F)** pH-dependent activity of the FK506 and CsA biosensors in the absence and presence of 200 nM ligand. **(G,H)** Temperature-dependent activity of the FK506 and CsA biosensors after incubation at the indicated temperatures and for the indicated times. Bars represent the mean ± SD from three technical replicates.

**Supplementary Figure 16.** Specificity analysis of the FK506 and CsA two-component biosensors. (**A)** Relative activity of the FK506 biosensor assay containing 50 nM BLA-CaM-CN-FKBP and 250 nM fB1-CaM-BP in the presence of DMSO (control), 200 nM FK506, 200 nM cyclosporine A (CsA) or 200 nM rapamycin. (**B-D)** Relative activities of CsA biosensor assays containing 35 nM BLA-CaM-CN-Cyp and 250 nM of the CaM-BP-fused binders cB6 (B), cB1 (C) or cG03 (D) under the same conditions. Error bars indicate standard deviations from two replicate measurements.

### Supplementary Text 3. Structural modelling explains the selectivity of fB1 for the CN-FKBP:FK506 complex

Despite numerous attempts, we were unable to obtain diffracting crystals of the developed ternary complexes. Therefore, we performed structural modelling of the CN-FKBP fusion and its complexes with selected binding domains. First, AlphaFold2 (AF2, pTM model) ^[29-30]^ was used to generate the structure of the apo form of CNA-CNB-FKBP linked by 9- and 32-aa GS linkers. As expected for long GS linkers, AF2 produced multiple interdomain arrangements rather than a single pose: Cα-based metrics (centroid distances CNA-CNB, CNA-FKBP and CNB-FKBP, and the clamp angle at CNA) grouped the five AF2 models into approximately 2-3 conformer families. Relative to the holo geometry, the apo ensemble was more open, with the CNB-FKBP distance 6-10 Å greater and the CNA clamp angle 14-19° larger, whereas CNA-CNB changed little (approximately -0.8 to -1.7 Å). The rank-1 apo model was used to seed ligand-bound predictions (Supplementary Figure 17A).

Subsequently, AlphaFold3 ^[31]^ was applied to the generated models in the presence of FK506 to produce five holo candidates (Supplementary Table 6). We ranked them by interface confidence (ipTM approximately 0.98 in the top models), overall fold (pTM approximately 0.846), FK506-pose consistency (pairwise ligand RMSD after protein Cα alignment), domain-clamp metrics and ligand-contact counts. To validate the model choice using experimental data, we compared each model with the crystallographic non-fusion holo complex 1TCO. AF3 sample_0 provided the best match: an FK506 heavy-atom RMSD of approximately 0.60 Å (after aligning FKBP Cα) and small deviations in clamp geometry (Δd CNA-CNB = -4.30 Å; Δd CNA-FKBP = -2.40 Å; Δd CNB-FKBP = -0.62 Å; Δ∠CNA = +4.55° relative to 1TCO). We therefore designated AF3 sample_0 as the reference holo model. Other solutions, however, converged on the same site and orientation of the conformation-selective binding domain (Supplementary Figure 17B).

We next used AlphaFold3 ^[31]^ to predict the binding mode of fB1 to CN-FKBP:FK506. Among the five predicted models, two distinct conformational classes emerged (conf-1 and conf-2; Supplementary Tables 7 and 8). Conf-1 comprises a single model with a slightly open clamp geometry, whereas conf-2 (four models) adopts a more closed geometry, characterised by reduced distances between CNA-FKBP and CNB-FKBP and a smaller interdomain angle at CNA (Supplementary Figure 17C). This 'closed-clamp' conformation positions FKBP closer to CN, resembling the holo-like clamped state of the complex. Conf-2 likely represents the physiologically relevant arrangement, in which FK506 binding draws FKBP towards CNA, forming a new composite epitope. fB1 engages primarily CNA and FKBP, with dominant contributions from CDR3, followed by CDR1 and CDR2 (Supplementary Table 7). In contrast, the apo complex, which likely presents a flexible ensemble of more open interdomain states, exhibits increased spacing and altered angles that prevent formation of the same interaction network.

In contrast to the CN-FKBP:FK506 system, modelling of the CN-Cyp:CsA fusion with AF3-Multimer produced only modest confidence scores (pTM approximately 0.66; ipTM approximately 0.54) and generated heterogeneous CN-Cyp orientations that did not reproduce the crystallographic arrangement observed in the non-fusion ternary complex (PDB 1MF8). Given these limitations, we did not pursue detailed analysis of the AF3-predicted CN-Cyp:CsA models or analyse the selectivity of cB6 for the CN-Cyp:CsA complex; further refinement will be required to obtain a reliable structural representation.

Supplementary Figure 17. Ligand-induced formation of an intramolecular composite interface and its recognition by selected FN3con binding domains. (A) Ribbon representation of the apo CN-FKBP complex comprising the catalytic subunit CNA (blue), the regulatory subunit CNB (green) and FKBP (red). (B) Holo CN-FKBP:FK506 complex highlighting FK506 (grey spheres) bound at the FKBP-CNA interface, inducing a clamped conformation that brings FKBP closer to CN. (C) Ribbon representation of holo CN-FKBP (grey) bound by four fB1 conf-2 models (coloured), which engage the FK506-induced composite epitope. FK506 is displayed as a molecular surface in element-specific colours.

**Supplementary Figure 18.** Semi-quantitative ELISA analysis of selected conformation-specific binders for FK506 and CsA detection. Dose-response curves were generated by immobilising biotinylated CN-FKBP or CN-Cyp on streptavidin-coated plates in the presence of increasing concentrations of the cognate ligand, followed by detection with the indicated FLAG-tagged FN3con binder and anti-FLAG-HRP. Absorbance at 450 nm was plotted against ligand concentration and fitted in GraphPad Prism to estimate the apparent dissociation constant (K_D,app_). For FK506, fB1-CaM-BP yielded a K_D,app_ of 1.605 ± 0.06 nM. For CsA, cB1-CaM-BP, cB6-CaM-BP, and cG03-CaM-BP yielded K_D,app_ values of 7.625 ± 0.18 nM, 12.95 ± 0.09 nM, and 11.6 ± 0.03 nM, respectively. Data is presented as the mean ± SD from three replicates. The corresponding goodness-of-fit values (R²) are shown in each panel.

**Supplementary Figure 19.** Quantification of CsA and FK506 using ELISA assays based on interactions between intramolecular CID systems and conformation-selective FN3con binders. (**A)** CsA calibration curve for an ELISA assay using CN-Cyp and 3Flag-cB1-CaM-BP as the binding reagents, yielding R² = 0.95 and a limit of detection of 0.13 nM. (**B**) FK506 calibration curve using CN-FKBP and 3Flag-fB1-CaM-BP, fitted by linear regression and yielding R² = 0.99 and an LOD of 0.05 nM. Data is presented as the mean ± SD from two independent experiments.

**Supplementary Figure 20.** Quantification of CsA and FK506 using ELISA assays based on the original CID systems. (A) Schematic representation of sandwich ELISA based on original CID. Streptavidin-coated plates are incubated with a pre-equilibrated mixture of biotinylated Cyp or FKBP (orange) and samples containing CsA or FK506. The resulting holo-complexes recruit the 3Flag (light blue) tagged calcineurin (cyan). Detection is performed using an HRP-conjugated anti-Flag antibody and TMB substrate. (B) ELISA-based quantification of CsA using surface-immobilised biotinylated Cyp and 3Flag-calcineurin in solution. (C) ELISA-based quantification of FK506 using surface-immobilised biotinylated FKBP and 3Flag-calcineurin for detection. Data are shown as the mean ± s.d. from two independent experiments.

**Supplementary Figure 21. Effect of whole human blood on ELISA assays based on the intramolecular CID complexes and their binding domains.** ELISA assays for CsA (left) and FK506 (right) were performed in the presence of 5 µL whole blood to evaluate matrix interference. Biotinylated CN-Cyp or CN-FKBP complexes were immobilised in streptavidin-coated wells, and detection was achieved using 3Flag-cB1 or 3Flag-fB1, respectively. Apparent dissociation constants (K_D_,app) were determined by nonlinear regression: 2.0 ± 0.19 nM for CsA and 1.04 ± 0.06 nM for FK506 (R² = 0.9903 and 0.9842, respectively), indicating minimal effects of blood components on assay performance. Data are shown as the mean ± s.d. from four replicates.

## MATERIALS AND METHODS

### Materials

Oligonucleotides were purchased from either Bioneer Pacific or Integrated DNA Technologies (IDT), as indicated in Supplementary Table 2. Chemicals were purchased from Sigma unless otherwise indicated. Human blood samples were collected under approval from the Coronial and Public Health Sciences Human Ethics Committee (CPHS-HEC), Pathology Queensland, Queensland Health (HREC/2026/CPHS/126933; HEC 26-14; EC00305), with a waiver of informed consent. Administrative review approval was provided by the Queensland University of Technology Office of Research Ethics and Integrity (HE-AdRev 2026-11316-30187).

### Expression and purification of biotinylated pseudo-allosteric fusion proteins controlled by FK506 and cyclosporine A

Genes encoding the designed pseudo-allosteric fusion proteins CN-FKBP and CN-Cyp, each containing an N-terminal His tag, AviTag and PreScission protease cleavage site, were codon-optimised, synthesised and cloned into the pET28a vector (Gene Universal). The protein sequences are provided in Supplementary Table 1. Proteins were co-expressed with BirA ligase for in *vivo* biotinylation as described previously ^[26]^. Briefly, sequence-verified expression plasmids and a BirA plasmid (MbI, Singapore) were co-transformed into BL21(DE3) cells and expressed in LB medium containing kanamycin and chloramphenicol. Cultures were supplemented with 25 mg/L biotin, induced with 0.3 mM IPTG, grown overnight at 18 °C and harvested by centrifugation. For the non-biotinylated version, the expression plasmid alone was used for transformation and protein production.

Cell pellets were resuspended in lysis buffer containing 50 mM sodium phosphate (pH 8.0), 300 mM NaCl, 20 mM imidazole, 1 mM AEBSF and DNase I, and disrupted using a CFII cell disrupter (Constant Systems, UK) at 26 kpsi. Following centrifugation at 20,000 rpm, the supernatant was collected and loaded onto a Ni-NTA HisTrap FF crude column operated using an ÄKTA PURE system (Cytiva). The binding buffer contained 50 mM sodium phosphate, 300 mM NaCl and 20 mM imidazole (pH 8.0), and the elution buffer contained 50 mM sodium phosphate , 300 mM NaCl and 500 mM imidazole (pH 8.0). Proteins eluted from the HisTrap column were further purified by size-exclusion chromatography using a HiLoad 16/600 Superdex 200 pg column (Cytiva). Before sample loading, the column was equilibrated with storage buffer containing 20 mM Tris-HCl (pH 7.2) and 100 mM NaCl. After size-exclusion chromatography, the peak corresponding to the expected size of the target protein was collected and concentrated using an Amicon Ultra-15 centrifugal filter unit (50 kDa MWCO). Purified proteins were aliquoted, snap-frozen in liquid nitrogen and stored at -80 °C.

The biotinylation efficiency of the expressed proteins was assessed using streptavidin Dynabeads M280 (Thermo Fisher Scientific). Purified protein samples (150 pmol) were incubated with pre-washed Dynabeads in PBS for 1 h at room temperature with gentle rotation. The beads were collected using a magnetic stand, and the unbound fraction was removed. The beads were then washed three times with PBS, and bound proteins were eluted by boiling in SDS-PAGE loading buffer. Bound and unbound fractions were analysed by SDS-PAGE and Coomassie staining. Biotinylation efficiency was determined by comparing the intensities of the protein bands in the bound and unbound fractions.

### Competitive binding assay for pseudo-allosteric fusion proteins

Competition assays were performed to estimate the binding affinities of CN-FKBP and CN-Cyp fusion proteins for their cognate ligands. For CN-FKBP, reactions contained 20 mM Tris-HCl (pH 7.2), 100 mM NaCl, 1 mM CaCl_2_, 250 nM M13 peptide, 1 µM PQQ, 2.5 nM cpGDH-CaM-FK506 sensor, 10 nM FK506 and varying concentrations of CN-FKBP fusion protein. For CN-Cyp, the same buffer composition was used, with 2.5 nM cpGDH-CaM-CsA sensor and 10 nM CsA replacing the corresponding sensor and ligand, and with varying concentrations of CN-Cyp fusion protein. All reactions were incubated at room temperature for 60 min. Biosensor activity was initiated by adding 20 mM glucose, 0.6 mM phenazine methosulfate (PMS) and 0.06 mM 2,6-dichlorophenolindophenol (DCPIP), and was monitored at 600 nm using a Cary UV-Vis spectrometer.

The slope of the initial linear phase of each reaction was used to determine *k*_obs_. The resulting *k*_obs_ values were plotted against the log concentration of unlabeled competitor (CN-FKBP or CN-Cyp) and fitted in GraphPad Prism using a one-site competitive binding model to determine K_i_, which was taken as equivalent to K_D_. The data were fitted using the equation: Y= (Top-Bottom)/(1+10^(X-LogEC_50_)) + Bottom, where X is the log concentration of unlabelled competitor, Top and Bottom are the fitted upper and lower plateaus. The logEC_50_ is determined from logEC_50_=log(10^logK_i_*(1+HotNM/Hot_KD,NM_)). For CN-FKBP, the concentration of labeled ligand (HotNM) and its dissociation constant (Hot,NM) were constrained to 2.5 nM and 1.4 nM, respectively. For CN-Cyp, these values were constrained to 2.5 nM and 0.3 nM, respectively. The K_i_ of the unlabeled competitor was calculated from the fitted logK_i_.

### mRNA display selection of conformation-selective CN-FKBP:FK506 binders

The mRNA display procedure was performed as previously described ^[26]^ , with modifications. Briefly, the naïve mRNA library was ligated to a puromycin linker (PuL) and purified using the Monarch® RNA Cleanup Kit (NEB). The mRNA-PuL conjugate (1 µM) was translated using a customised PUREfrex^®^ 1.0 kit lacking RF1, RF2, RF3 and RRF (COSMO BIO Co. LTD.) in a total volume of 200 µL at 37 °C for 50 min. The translation reaction was subjected to reverse transcription using the SuperScript™ III First-Strand Synthesis System (Thermo Fisher Scientific) with primer 64 at 42 °C for 20 min. EDTA (final concentration, 10 mM) was added, and the sample was diluted tenfold with PBST and centrifuged at 21,000 × g at 4 °C for 5 min. The supernatant was incubated with 40 µL of CN-FKBP-saturated Dynabeads™ MyOne™ Streptavidin C1 (Thermo Fisher Scientific) at 20 °C for 30 min to remove binders to the apo protein. The supernatant was then incubated with 40 µL of CN-FKBP:FK506-saturated Dynabeads C1 for 20 min. Beads were sequentially washed with 1 mL of each of the following: (i) PBST + 1 µM FK506, (ii) PBST + 1 µM FK506 + 360 mM NaCl, (iii) PBST + 500 pmol CN-FKBP and (iv) PBST + 1 µM FK506. Washed beads were transferred to a new low-binding microcentrifuge tube (Costar®, #3207), resuspended in 1 mL PCR buffer (10 mM Tris-HCl, pH 8.4, 100 mM KCl, 0.1% Triton X-100, 2% DMSO and 2 mM MgSO_4_), heated at 95 °C for 5 min, cooled and subjected to PCR after addition of dNTPs (0.2 mM), PfuX7 DNA polymerase ^[32]^ (2 nM) and primers 54 and 64 (0.25 µM each). PCR conditions were 95 °C for 1 min; eight cycles of 95 °C for 30 s, 68 °C for 30 s and 72 °C for 30 s; and a final extension at 72 °C for 2 min. PCR products were purified using the Wizard® SV Gel and PCR Clean-Up System (Promega) and eluted in 50 µL RNase-free water.

Purified cDNA was used for round 2 selection by performing *in vitro* transcription, DNase I treatment and PuL ligation as described previously ^[26]^. The mRNA-PuL conjugate was eluted in 12 µL RNase-free water. For rounds 2-6, reaction volumes, bead types and washing stringency were adjusted to increase selection pressure. Translation volumes were reduced to 40 µL in R2 and 25 µL in R3-R5. Counter-selections used either CN-FKBP-saturated C1 Dynabeads (R2, R3 and R6) or CN-FKBP-saturated NeutrAvidin magnetic particles (Spherotech) (R5). Positive selections were performed by incubating the supernatant with 10 µL of CN-FKBP:FK506-saturated beads for 20 min (R2-R4) or 15 min (R5-R6). The number of washes increased progressively: four washes (R2-R3), six washes (R4), eight washes (R5) and ten washes (R6). Each two-wash increment included one wash with PBST + 1 µM FK506 and one wash with PBST + 1 µM FK506 + 360 mM NaCl.

### mRNA display selection of conformation-selective CN-Cyp:CsA binders

The selection campaign for conformation-selective binders against the CN-Cyp:CsA complex followed the FK506-selection protocol with minor modifications. Translation volumes were 100 µL for R1 and 25 µL for R2-R5. Counter-selection in R1, R2 and R4 was performed using CN-Cyp-saturated C1 Dynabeads, whereas R3 and R5 used CN-Cyp-saturated NeutrAvidin beads. Positive selection in R1, R2 and R4 was performed using CN-Cyp:CsA-saturated C1 Dynabeads, whereas R3 and R5 used CN-Cyp:CsA-saturated NeutrAvidin beads. In R1, positive-selection beads were washed twice with 1 mL PBST + 1 µM cyclosporine, followed by 1 mL PBST + 200 pmol CN-Cyp and a final wash with 1 mL PBST + 1 µM cyclosporine. The number of washes in R2 and R3 was increased to six, with two additional washes in PBST + 1 µM CsA + 360 mM NaCl. No RNA transcripts were recovered after R3, suggesting that the R2 washing conditions may have been too stringent and eliminated potential binders. To address this, R3 selection was repeated using a mixed input comprising 40% R2-output mRNA and 60% R1-output mRNA. We successfully recovered putative binders and subjected them to R4 and R5 selection, each involving two rounds of counter-selection (two 22 min incubations) followed by a single positive selection (15 min incubation). Washing stringency was further increased to eight washes in R4 and ten washes in R5.

### ELISA-based analysis of FN3con-binder enrichment across selection rounds

To assess binder enrichment in each selection round, ELISA was performed using a Maxisorp plate (#442404, Thermo Fisher Scientific) coated with streptavidin (Sigma) at 10 µg/mL in PBS and incubated overnight at room temperature. Plates were washed three times with PBST (PBS containing 0.1% Tween). Wells in columns 2-6 and 8-12 were incubated with control protein (10 µg/mL in PBS) and protein-ligand complex (10 µg/mL protein + 2.5 µM ligand in PBS), respectively, and blocked with 2% MPBS (PBS containing 2% skim milk) for 1 h. Columns 1 and 7 were incubated with PBS and served as negative controls.

In parallel, cDNAs from the indicated selection rounds were translated into protein using the PUREfrex^®^ kit at 30 °C for 3 h and blocked with 2% MPBS. After removal of the blocking buffer from the ELISA plate, translation reactions were added to columns 2-5 and 8-11 in tenfold serial dilutions. Columns 6 and 12 were not incubated with translation reaction and served as negative controls. After a 1 h incubation, the plate was washed three times with PBST and then incubated with 100 µL anti-FLAG-HRP (1:5000 dilution; Sigma A8592) for 1 h. Plates were washed again, and 100 µL TMB solution (#34028, Thermo Fisher Scientific) was added to each well for 10 min or until adequate colour development. The reaction was stopped with 1 M sulfuric acid, and absorbance was measured at 450 nm (BioTek Synergy Neo2).

### NGS-based analysis of selection outcomes

cDNAs from the naïve FN3con library (R0) and each selection round for CN-FKBP:FK506 and CN-Cyp:CsA binders were PCR-amplified to add adapter sequences using 1 ng/µL template and 0.25 µM each of primers 219 and 221. PCR was performed using a three-step program (annealing at 60 °C; 12 cycles), and the products (50 µL) were purified with 40 µL Agencourt AMPure XP beads and eluted in 40 µL RNase-free water. Index sequences were then incorporated into each cDNA sample using the Nextera Index Kit v2 (Illumina), followed by AMPure XP bead purification. Indexed libraries were quantified using a Qubit fluorometric assay (Thermo Fisher Scientific) and assessed for size distribution and purity using an Agilent fragment analyser. cDNA sequencing was performed at the CARF facility (QUT) on an Illumina NovaSeq platform using the MiSeq Reagent Kit v2 (500 cycles).

NGS data were processed using custom Python scripts. Paired-end reads from R1 and R2 were merged with NGmerge in dovetail mode (the -d flag), which also runs in overlap mode and selects the best merge for each read. On average, 88% of the reads were successfully stitched together. The most frequent read length was 285 bp, as expected for FN3con. The amplicon sequence contained three randomised segments interspersed among four fixed sequences (N-to-C order: TDVTSTSVTLSW, GYRVEYREAGGEWKEVTV, YTVTGLKPGTEYEFRVRAV and SSVSVTT); our code parsed the sequences between adjacent fixed sequences as the randomised segments. Frequencies were then tabulated to enable ratio calculations and analysis of inter-round changes in the parsed randomised segments.

Custom R scripts were used to plot changes in the parsed randomised segments across panning rounds. Trends were plotted as normalised frequencies ('frequency per million') to account for differences in sequencing depth among libraries. For computational efficiency, particularly for correlation analyses in which processing time is proportional to the square of the number of unique peptides, trends were plotted for peptides in the top 5% by frequency. Heatmaps of peptide-frequency similarity across panning campaigns and rounds were based on pairwise Pearson correlations (*r* values) and clustered using the default settings of the pheatmap library (v1.0.13; <https://github.com/raivokolde/pheatmap>).

The evolution of normalised frequencies across panning rounds was visualised using principal component analyses (PCAs) and bump charts. Overall trends were visualised with PCAs: colours indicate the target used for panning, and lines connect libraries across successive panning rounds to show changes in frequency across PCA space. Specific trends were visualised with bump charts. To determine whether peptides with similar motifs were similarly enriched across panning rounds, sequences were grouped into five higher-order clusters using the PAM30 distance matrix. Clustering was performed with a custom Python script executed from R using the reticulate library. Because the clustering algorithm is computationally expensive and the vast majority of peptides are not enriched, only the 100 most frequent randomised segments from each panning round were retained for this analysis. For visual clarity, the randomised-segment sequences from the first and final panning rounds are shown in full; the other sequences are hidden.

### Cloning of cDNA pools into the CaM-BP fusion-expression vector

The cDNA pool from the final round of each selection was PCR-amplified with primers 215 and 226 to introduce overlap regions. PCR products were separated on a 2% agarose gel and purified using the Wizard® SV Gel and PCR Clean-Up System (Promega). The purified PCR products and BamHI/SalI-double-digested pET28a-His-insertion-CaM-BP vector ^[26]^ were assembled using Gibson Assembly Master Mix (NEB) by incubation at 50 °C for 1 h. The assembled products were transformed into *E. coli* TOP10 competent cells (Thermo Fisher Scientific) and plated on two LB agar plates: one for picking individual colonies and the other for pooling all colonies for plasmid minipreparation, followed by transformation into BL21(DE3) cells for expression.

Ninety-five individual colonies from each selection, derived from either TOP10 or BL21 cells, were picked into two separate 96-well plates. One plate contained 200 µL LB medium with 50 µg/mL kanamycin per well, whereas the other contained 40 µL UltraPure™ DNase/RNase-Free Distilled Water (#10977023, Thermo Fisher Scientific) per well. The LB plate was cultured at 37 °C for 10-14 h, supplemented with 20% glycerol and stored at -80 °C. The water plate was heated at 95 °C for 8 min and used as the template for colony PCR with primers 222 and 223 (annealing at 50 °C; 30 cycles). CaM-BP constructs were sequenced by Macrogen using primer 222.

### Recombinant expression and purification of potential FN3con binders

Plasmids containing potential binders identified by sequence analysis were isolated from TOP10 cells using the QIAprep Spin Miniprep Kit (QIAGEN) and transformed into *E. coli* BL21(DE3) competent cells (Thermo Fisher Scientific). Transformed BL21(DE3) cells, or BL21(DE3) clones identified by the biosensor assay, were cultured in LB broth containing kanamycin at 37 °C with shaking. When the optical density (OD_600_) reached 0.8, protein expression was induced with 0.3 mM IPTG, followed by overnight incubation at 18 °C. Cells were lysed, and proteins were purified using HisTrap columns as described for the pseudo-allosteric fusion proteins. Eluted proteins were dialysed against 20 mM Tris-HCl (pH 7.2) and 100 mM NaCl, aliquoted, snap-frozen in liquid nitrogen and stored at -80 °C.

### Biosensor assay

The functionality of the BLA-CaM-CN-FKBP and BLA-CaM-CN-Cyp proteins was assessed at concentrations of 25 and 50 nM, respectively, using a β-lactamase enzymatic assay. Reactions were prepared in buffer containing 20 mM Tris-HCl (pH 7.2), 100 mM NaCl, 1 mM CaCl_2_ and 200 nM M13 CaM-BP and incubated for 30 min. The assay was initiated by adding 50 µM UW154, and absorbance was monitored at 520 nm using a Neo2 plate reader.

For screening FN3con-CaM-BP colonies against FK506, assays were performed in 96-well plates containing 20 mM Tris-HCl (pH 7.2), 100 mM NaCl, 1 mM CaCl_2_, 35 nM BLA-CaM-CN-FKBP and 30 µL clarified supernatant from BL21(DE3) colonies expressing FN3con-CaM-BP constructs (after three freeze-thaw cycles), in the presence or absence of 1µM FK506. After a 30 min incubation, UW154 substrate was added, and the reaction was monitored as described above. Screening against CsA followed the same procedure but used 35 nM BLA-CaM-CN-Cyp and 1µM CsA.

Ligand-titration assays were performed to determine the apparent K_D_ values of the biosensors for their respective ligands. For FK506, reactions contained 20 mM Tris-HCl (pH 7.2), 100 mM NaCl, 1 mM CaCl_2_, 50 nM BLA-CaM-CN-FKBP and 250 nM fB1-CaM-BP, with FK506 concentrations ranging from 0 to 500 nM. After a 30 min incubation, the reaction was initiated by adding UW154 substrate and monitored as described above. For CsA, the assay was performed in the same buffer using 35 nM BLA-CaM-CN-Cyp and 175 nM cB6-CaM-BP, with CsA concentrations ranging from 0 to 500 nM. Following a 30 min incubation, the reaction was initiated with UW154 substrate and monitored under identical conditions.

The slope of the initial linear phase of each reaction was recorded as k_obs_. The k_obs_ values were plotted against ligand concentration and fitted in GraphPad Prism to calculate K_D_ using the equation Y = B_max_ × X/(K_D_ + X) + NS × K_D_/(K_D_ + X) + Background, where B_max_ is the maximum specific binding, NS is the slope of non-specific binding and Background is non-specific binding in the absence of ligand. Dynamic range was calculated as the ratio of k_obs_ at a saturating ligand concentration to k_obs_ in the absence of ligand. The limit of detection (LOD) was calculated from the linear range of the titration curve as LOD = 3 × SD/S, where SD is the standard deviation of the response and S is the slope of the calibration curve.

To test the pH tolerance of the FK506 biosensor, we incubated 50 nM BLA-CaM-CN-FKBP and 250 nM fB1-CaM-BP in buffers spanning pH 2-10, in the absence or presence of 200 nM FK506, for 30 min. UW154 substrate was then added, and enzymatic activity was recorded at 520 nm using a Neo2 plate reader. A similar assay was performed for the CsA biosensor using 35 nM BLA-CaM-CN-Cyp and 175 nM cB6-CaM-BP in the absence or presence of 200 nM CsA.

To test biosensor thermostability, we incubated 2 µM BLA-CaM-CN-FKBP and 10 µM fB1-CaM-BP at the indicated temperatures for the indicated periods. We then performed the standard biosensor assay, as described above, in the absence or presence of 200 nM FK506. After a 30 min incubation, UW154 substrate was added, and biosensor activity was monitored at 520 nm. A similar assay was performed for the CsA biosensor.

### ELISA-based assay for FK506 and CsA quantification

A Maxisorp plate (Thermo Fisher Scientific) was coated with streptavidin (10 µg/mL in PBS) and incubated overnight at room temperature. The plate was washed three times with PBS and incubated with biotinylated CN-FKBP or CN-Cyp (10 µg/mL) together with either defined concentrations of FK506 or CsA respectively, or whole blood samples. After a 1 h incubation, the plate was washed three times with PBST and then blocked with 2% MPBS for 1 h. After decanting the blocking solution, the corresponding purified Flag-tagged binder (200 nM in 2% MPBS) was added and incubated for 1 h, followed by three PBST washes. Wells were then incubated with 100 µL anti-FLAG-HRP (1:5000 dilution; Sigma A8592) for 1 h and washed four times with PBST. TMB solution (100 µL; #34028, Thermo Fisher Scientific) was added, and the reaction was stopped with 1 M sulfuric acid after 10 min or once sufficient blue colour had developed. Absorbance at 450 nm was measured using a Neo2 plate reader.

For matrix-interference experiments, 5 µL of whole blood was used for both FK506 and CsA assays, representing the maximum blood volume tested for matrix interference. For quantification of patient samples, 4 µL of whole blood was used for FK506 measurements, whereas 0.7 µL was used for CsA measurements. Standard curves were generated using varying concentrations of the corresponding drug and analysed in GraphPad Prism using a one-site total least-squares model to estimate the apparent K_D_. The limit of detection (LOD) was calculated from the linear range of the titration curve as described above.

### Biolayer interferometry assay

Biolayer interferometry (BLI) binding data were acquired using an Octet RED8 system (ForteBio) equipped with eight streptavidin (SA) biosensor channels. All assays were performed in PBS containing 0.05% Tween-80 and 1% BSA. Each kinetic assay comprised equilibration in assay buffer for 120 s, loading of biotinylated CN-FKBP or CN-Cyp fusion protein in either *apo* or *holo* form for 300 s, a second equilibration in assay buffer for 120 s, association with binders for 300 s and dissociation in assay buffer for 300 s.

For *holo*-protein assays, sensors were immobilised with 10 µg/mL biotinylated CN-FKBP or CN-Cyp pre-saturated with 2 µM of the respective ligand, followed by titration with FN3con binders at 400, 200, 50, 25 and 0 nM. For *apo*-protein assays, sensors were loaded with 10 µg/mL biotinylated CN-FKBP or CN-Cyp in the absence of ligand and titrated with FN3con binders at 2, 1, 0.5 and 0 µM. Data were processed and analysed using ForteBio Data Analysis 9.0 software.

### Pull-down assay

Streptavidin M280 Dynabeads (20 µL per reaction; Thermo Fisher Scientific) were incubated with 20 µL of 4 µM biotinylated CN-FKBP or CN-Cyp, 12 µM FN3con binding protein and either 20 µM cognate ligand or no ligand (control) in PBS. The mixture was rotated at 1,250 rpm for 1 h at room temperature. Beads were washed three times with PBST (PBS + 0.1% Tween-20) to remove unbound proteins. Bound proteins were eluted by heating in SDS-PAGE loading buffer and analysed by SDS-PAGE.

### Calculation of clamp-geometry metrics for the fusion proteins

Domains (residue indices in the fusion):
CNA = 44-428, CNB = 438-605 and FKBP = 637-743

d(CNA-FKBP) and d(CNB-FKBP)

These are Euclidean distances between domain centroids, where each centroid is the average of all Cα coordinates within the residue range of that domain. They describe how close FKBP is to CNA or CNB.

The centroid of domain X was calculated as follows:

$$C_{X}=\frac{1}{N}\sum_{i\in\text{res}(X)} r_{i}^{C\alpha}$$

The distances were calculated as follows:

$$d(CNA,FKBP)=\parallel C_{\mathrm{CNA}}-C_{\mathrm{FKBP}}\parallel$$

$$d(CNB,FKBP)=\parallel C_{\mathrm{CNB}}-C_{\mathrm{FKBP}}\parallel$$

Smaller values indicate a tighter clamp, with the domains drawn closer together.

Angle at CNA (degrees)

The internal angle at the CNA centroid is formed by the vectors from CNA to CNB and from CNA to FKBP:

The vectors were defined as follows:

$$v_{1}=C_{\mathrm{CNB}}-C_{\mathrm{CNA}}$$

$$v_{2}=C_{\mathrm{FKBP}}-C_{\mathrm{CNA}}$$

The angle (in degrees) was calculated as follows:

$$\theta_{\mathrm{CNA}}={cos}^{-1}\text{ ⁣}(\frac{v_{1}\cdot v_{2}}{\parallel v_{1}\parallel\text{ }\parallel v_{2}\parallel})$$

A smaller angle indicates that CNB and FKBP lie more nearly on the same side of CNA, corresponding to a more closed clamp; a larger angle indicates a more open arrangement.

### Classification of the five CN-FKBP:FK506:fB1 models into conformational clusters

1. Reduce each model to a three-number 'clamp-geometry' vector
For each model m, we calculated domain Cα centroids using the fusion-residue ranges (CNA 44-428; CNB 438-605; FKBP 637-743) and then calculated:

$$d_{AF}^{(m)}=\parallel\mathbf{C}_{\mathrm{CNA}}-\mathbf{C}_{\mathrm{FKBP}}\parallel$$

$$d_{BF}^{(m)}=\parallel\mathbf{C}_{\mathrm{CNB}}-\mathbf{C}_{\mathrm{FKBP}}\parallel$$

$\theta_{\mathrm{CNA}}^{(m)}={cos}^{-1}\text{ ⁣}(\frac{(\mathbf{C}_{\mathrm{CNB}}-\mathbf{C}_{\mathrm{CNA}})\cdot(\mathbf{C}_{\mathrm{FKBP}}-\mathbf{C}_{\mathrm{CNA}})}{\parallel\mathbf{C}_{\mathrm{CNB}}-\mathbf{C}_{\mathrm{CNA}}\parallel\text{ }\parallel\mathbf{C}_{\mathrm{FKBP}}-\mathbf{C}_{\mathrm{CNA}}\parallel})$(angle expressed in degrees)

Thus, each model is represented by the point $g^{(m)}=(d_{AF}^{(m)},\text{ }d_{BF}^{(m)},\text{ }\theta_{\mathrm{CNA}}^{(m)})$.

2. Measure the differences among models in this three-dimensional space
We calculated all pairwise Euclidean distances:

$$D_{ij}=\sqrt{(d_{AF}^{(i)}-d_{AF}^{(j)})^{2}+(d_{BF}^{(i)}-d_{BF}^{(j)})^{2}+(\theta^{(i)}-\theta^{(j)})^{2}}.$$

If ${max}_{i,j}D_{ij}<\varepsilon$, with $\varepsilon=2.0$ in the mixed Å/° units, the models were classified as a single conformer.

In our data, $maxD_{ij}\approx3.81$, indicating more than one group.

3. Identify the most widely separated pair and split by the nearest centroid (one-pass, two-way split)

The most widely separated pair $(i^{\text{\textbackslash*}},j^{\text{\textbackslash*}})=argmaxD_{ij}$was identified as follows:

We used $g^{(i^{\text{\textbackslash*}})}$and $g^{(j^{\text{\textbackslash*}})}$as provisional cluster centroids.

Each model m was assigned to the closer centroid using the nearest-centroid rule in this three-dimensional space.

4. Check within-cluster spread and determine whether a third cluster is required

For each cluster k, we calculated the maximum internal spread $\max D_{pq}$among members p and q.

If both cluster spreads were no greater than $\varepsilon$, two classes were retained.

If a cluster still had a spread greater than $\varepsilon$ and contained enough points, that cluster was split once more using the same farthest-pair and nearest-centroid rule to produce three classes.

In our case, both clusters were sufficiently compact after the first split, so two conformers were retained.

### **References**

[26]	Z. Cui, C. E. Ayva, Y. J. Liew, Z. Guo, R. Mutschler, B. Dreier, M. M. Fiorito, P. Walden, C. B. Howard, F. Ely, A. Pluckthun, C. Pretorius, J. P. Ungerer, A. M. Buckle, K. Alexandrov, mRNA Display Pipeline for Protein Biosensor Construction, *ACS Sens* **2024**, *9*, 2846-2857.

[27]	Z. Guo, O. Smutok, C. E. Ayva, P. Walden, J. Parker, J. Whitfield, C. E. Vickers, J. P. J. Ungerer, E. Katz, K. Alexandrov, Development of epistatic YES and AND protein logic gates and their assembly into signalling cascades, *Nat Nanotechnol* **2023**.

[28]	Z. Guo, O. Smutok, C. E. Ayva, P. Walden, J. Parker, J. Whitfield, C. E. Vickers, J. P. J. Ungerer, E. Katz, K. Alexandrov, Development of epistatic YES and AND protein logic gates and their assembly into signalling cascades, *Nat Nanotechnol* **2023**, *18*, 1327-1334.

[29]	J. Jumper, R. Evans, A. Pritzel, T. Green, M. Figurnov, O. Ronneberger, K. Tunyasuvunakool, R. Bates, A. Zidek, A. Potapenko, A. Bridgland, C. Meyer, S. A. A. Kohl, A. J. Ballard, A. Cowie, B. Romera-Paredes, S. Nikolov, R. Jain, J. Adler, T. Back, S. Petersen, D. Reiman, E. Clancy, M. Zielinski, M. Steinegger, M. Pacholska, T. Berghammer, S. Bodenstein, D. Silver, O. Vinyals, A. W. Senior, K. Kavukcuoglu, P. Kohli, D. Hassabis, Highly accurate protein structure prediction with AlphaFold, *Nature* **2021**, *596*, 583-589.

[30]	M. Mirdita, K. Schutze, Y. Moriwaki, L. Heo, S. Ovchinnikov, M. Steinegger, ColabFold: making protein folding accessible to all, *Nat Methods* **2022**, *19*, 679-682.

[31]	J. Abramson, J. Adler, J. Dunger, R. Evans, T. Green, A. Pritzel, O. Ronneberger, L. Willmore, A. J. Ballard, J. Bambrick, S. W. Bodenstein, D. A. Evans, C. C. Hung, M. O'Neill, D. Reiman, K. Tunyasuvunakool, Z. Wu, A. Zemgulyte, E. Arvaniti, C. Beattie, O. Bertolli, A. Bridgland, A. Cherepanov, M. Congreve, A. I. Cowen-Rivers, A. Cowie, M. Figurnov, F. B. Fuchs, H. Gladman, R. Jain, Y. A. Khan, C. M. R. Low, K. Perlin, A. Potapenko, P. Savy, S. Singh, A. Stecula, A. Thillaisundaram, C. Tong, S. Yakneen, E. D. Zhong, M. Zielinski, A. Zidek, V. Bapst, P. Kohli, M. Jaderberg, D. Hassabis, J. M. Jumper, Accurate structure prediction of biomolecular interactions with AlphaFold 3, *Nature* **2024**, *630*, 493-500.

[32]	M. H. Norholm, A mutant Pfu DNA polymerase designed for advanced uracil-excision DNA engineering, *BMC Biotechnol* **2010**, *10*, 21.
